# Non-Destructive Detection of Damaged Strawberries after Impact Based on Analyzing Volatile Organic Compounds

**DOI:** 10.3390/s22020427

**Published:** 2022-01-06

**Authors:** Yang Cao, Yuchen Zhang, Menghua Lin, Di Wu, Kunsong Chen

**Affiliations:** 1 The Rural Development Academy, Zhejiang University, Hangzhou 310058, China; caoy@zju.edu.cn; 2 College of Agriculture & Biotechnology, Zhejiang University, Hangzhou 310058, China; 0920569@zju.edu.cn (Y.Z.); 21616066@zju.edu.cn (M.L.); akun@zju.edu.cn (K.C.); 3 Zhejiang Provincial Key Laboratory of Horticultural Plant Integrative Biology, Zhejiang University, Hangzhou 310058, China; 4 The State Agriculture Ministry Laboratory of Horticultural Plant Growth, Development and Quality Improvement, Zhejiang University, Hangzhou 310058, China; 5 Digital Cold Chain and Logistics Research Center, Zhongyuan Institute, Zhejiang University, Zhengzhou 450000, China

**Keywords:** strawberry, impact damage, electronic-nose, GC-MS, volatile organic compound

## Abstract

Strawberries are susceptible to mechanical damage. The detection of damaged strawberries by their volatile organic compounds (VOCs) can avoid the deficiencies of manual observation and spectral imaging technologies that cannot detect packaged fruits. In the present study, the detection of strawberries with impact damage is investigated using electronic nose (e-nose) technology. The results show that the e-nose technology can be used to detect strawberries that have suffered impact damage. The best model for detecting the extent of impact damage had a residual predictive deviation (RPD) value of 2.730, and the correct rate of the best model for identifying the damaged strawberries was 97.5%. However, the accuracy of the prediction of the occurrence time of impact was poor, and the RPD value of the best model was only 1.969. In addition, the gas chromatography–mass spectrophotometry analysis further shows that the VOCs of the strawberries changed after suffering impact damage, which was the reason why the e-nose technology could detect the damaged fruit. The above results show that the mechanical force of impact caused changes in the VOCs of strawberries and that it is possible to detect strawberries that have suffered impact damage using e-nose technology.

## 1. Introduction

Strawberry is a fruit that is loved by a wide range of consumers due to its juicy taste, unique tangy-sweet taste, and wealth of nutrients, vitamins, and minerals. Nevertheless, strawberry is soft and, therefore, very vulnerable to mechanical damage during the post-harvest supply chain. Impact, compression, and vibration are the main mechanical forces that can cause fruit damage [1,2]. When fruit is mechanically damaged, its physiological metabolism becomes abnormal, such as quick softening [3], water losses [4], and oxidation browning [5]. Moreover, the mechanically damaged fruit is also more susceptible to infection by bacteria and fungi, which can easily cause fruit decay and affect fruit safety [6]. Therefore, mechanically damaged fruit is not suitable for further storage and sale, which, in turn, leads to a significant decline in the market value of the product.

In a competitive market, accurate and timely detection of whether the fruit is damaged by mechanical forces or not is important to provide information for post-harvest storage, transportation, and retail sale to optimize storage, transportation, and sales strategies. Retailers, sellers, and consumers usually identify the damaged fruit with the naked eye. For inspection purposes, digital vernier calipers are often used to measure the bruised area of fruits. Nevertheless, both visual inspection and the evaluation with digital verniers are inefficient, subjective, tedious, labor-intensive, and selected [7,8]. On the other hand, compared with manual inspection, spectroscopic and imaging technologies such as visible and near-infrared spectroscopy and hyperspectral imaging are non-invasive and allow for more rapid detection and have been used to detect the mechanical damage of fruits [9,10]. Nevertheless, the fruits are usually packaged in cardboard boxes or plastic boxes and then placed in a dark environment during supply chains, and, in some cases, the fruits are even wrapped with paper or foam nets, resulting in the inability to detect mechanical damage by spectroscopic and imaging methods. Moreover, when the fruit is damaged by impact, there might be no apparent bruise on the surface of the fruit immediately; when the bruise becomes evident, in most cases, the fruit is already placed in the packages, which makes the visual or imaging detection impossible.

Volatile organic compounds (VOCs) are a key indicator for fruit quality assessment and are also an important consideration for customers [11]. Previous studies have shown that VOCs of fruit can change after the fruit is damaged [12,13]. Therefore, analyzing the VOCs of fruit provides a possible way to detect the fruit with mechanical damage. The electronic nose (e-nose) is a widely used bionic olfactory system and a widely used technology for detecting VOCs [14,15,16]. It has obtained much attention because of its advantages of rapid and straightforward operation, non-destructive detecting, and cost-effectiveness. Previously, the e-nose systems have been used to classify fruit grades and predict fruit quality in peach [17], apple [18], apricot [19], mandarin [20], Goji berries [21], sweet cherries [22], and mango [23]. Especially for strawberries, the e-nose shows its capability of determining the freshness of strawberries [24], detecting fungal disease in strawberries in the early stage [25], and characterizing processed strawberry juices [26]. However, to the best of our knowledge, e-nose technology is seldom used to study the detection of mechanical damage to fruits. Moreover, it should be noted that many previous works on investigating fruit quality using e-nose technology are based on a destructive process of sampling, in which the fruit was cut or sliced to obtain more VOCs [27,28]. However, in the actual fruit supply chain, the detection of VOCs must be carried out on the intact fruit, and no destructive sampling can be carried out.

The main objective of this study is to use e-nose technology to achieve rapid and non-destructive detection of damaged strawberries and investigate the characteristic VOCs of strawberries after suffering from impact damage. The outcome of the study is meant to remove the damaged strawberries and further optimize post-harvest strategies and reduce economic losses.

## 2. Materials and Methods

### 2.1. Sample Preparation

Fresh strawberries (*Fragaria × ananassa* Duch.) were obtained from a local fruit store nearby the laboratory at the Institute of Fruit Science, Zhejiang University, and transported to the laboratory immediately. The fruit was harvested in the morning on the day of purchase. In order to ensure that the extent of mechanical damage to the fruit is controllable, strawberries with a uniform commercial maturity and no mechanical damage or disease were selected for further experimental detection. The strawberries were randomly divided into four groups (I, II, III, and IV), and each group contained 24 samples. Group I was used as the control group (no impact treatment). To obtain different extents of impact damage, the fruits of Group II, III, and IV were subject to free-fall onto a steel plate from the heights of 20, 40, and 60 cm, respectively. These heights are all settings of the three heights commonly used in studies to simulate the extent of fruit impact damage [29,30,31]. Each fruit was impacted on its side. All impact treatments were carried out at 15 °C and a humidity of 90–95% in the afternoon on the day of harvest. After the treatment, the strawberries were stored in cold storage at 15 °C and 90–95% humidity to simulate the storage environment in practice. It should be noted that the samples in the control group were used for model calibration as well as the samples in the other groups. This is because, in the supply chain, not every fruit will suffer mechanical damage. Therefore, to calibrate the detection models of mechanical damage, it is necessary to consider those fruits that did not suffer mechanical damage in the sample set used for model calibration.

### 2.2. E-Nose Instrument and Data Measurement

The e-nose system used was FOX 4000 (Alpha MOS, Toulouse, France), which is equipped with 18 metallic oxide gas sensors in three sensor chambers. VOCs of strawberries in each group were acquired at 0 h (before impact) and 4, 8, and 24 h after impact. Before extracting VOCs, each fruit sample was placed in a 100 mL glass beaker. Parafilm PM996 (Pechiney Plastic Packaging, Menasha, WI, USA) was used to seal the beaker for 1 h to equilibrate the headspace inside. The whole process of generating headspace gas was carried out at 15 °C to simulate the actual condition in practice. For the acquisition of VOCs, 2 mL of headspace gas was injected into the Fox 4000 system. The gas was then pumped into the sensor array with a constant rate of 150 mL·min^−1^. The entire detection progress of each sample lasted 6 min, including 120 s for the measurement phase and 240 s for the clean phase.

### 2.3. Sample Sets and Feature Matrix

In order to verify whether the e-nose technology could be used to detect strawberries that have suffered mechanical damage due to impact, a total of three types of models was developed in the present study. The first type of model was the one for the prediction of the extent of impact damage to strawberries falling from different heights, including 0, 20, 40, and 60 cm. Therefore, the dependent variable for modeling was the extent of impact damage, which included: 0, 20, 40, and 60 cm. The second type of model was the one for the identification of strawberries with impact damage. In this type of model, there were two types of samples, i.e., fruits that have suffered impact damage and fruits that have not suffered impact damage. Therefore, the dependent variable for modeling was whether the fruits have suffered impact damage, including: yes (1) and no (−1). The third type of model was the one for the prediction of the time of occurrence of the impact on the fruit. In the present study, the time between impact occurrence and e-nose data measurement (the time after the occurrence of the impact on the fruit) was used to represent the time of occurrence of the impact on the fruit. Specifically, there were four kinds of times to be predicted, which were 0, 4, 8, and 24 h after impact. Since the VOCs of the fruit will change after being mechanically damaged, the present work analyzed the VOCs of the strawberry fruit and established quantitative models between the VOC data and the time between impact occurrence and e-nose data measurement so as to predict when the strawberry was impacted. Therefore, the dependent variable for modeling was the time of the occurrence of the impact on the fruit, including: 0, 4, 8, and 24 h. For a specific impact extent and a specific post-impact storage time, there were 24 samples. For each model sample set, 75% of the samples were randomly selected as the calibration set, and 25% of the samples were selected as the prediction set.

A total of two major categories of variable sets were used as inputs to establish the chemometric models in this study. One category included all 18 sensors, each measuring 121 s (121 variables), so there were 2178 variables in total (EN_All_). The other category included some characteristic variables of the 18 sensors, such as the response data at the 10th, 20th, 40th, 60th, 80th, 100th, and 120th seconds, which were called EN_10_, EN_20_, EN_40_, EN_60_, EN_80_, EN_100_, and EN_120_, respectively; the sum of the response curve values, which was called EN_Sum_; the maximum value of the response curve, which was called EN_Max_; the minimum value of the response curve, which was called EN_Min_; and the difference between the maximum and minimum values of the response curve, which was called EN_Diff_. Extracting the signal at different time points of the e-nose signal acquisition process is a commonly used method to select the e-nose signal for modeling. Additionally, EN_Sum_, EN_Max_, EN_Min_, and EN_Diff_ are frequently used for the modeling of e-nose data, including in studies such as the prediction of the ripeness of kiwifruit [32] and the detection of the quality of pecans [33]. The above-mentioned variables were used separately to establish the models to determine the best characteristic variable. Only one characteristic variable was used in each model, and because there were 18 sensors, there were 18 variables in total for each model, based on the characteristic variable. In general, for all types of modeling, both EN_All_ and characteristic variables were used separately.

### 2.4. Multivariate Data Analysis and Model Evaluation

The regression models were established using partial least squares regression (PLSR) and least squares support vector machine (LS-SVM) algorithms, respectively. PLSR is a multivariate statistics-based linear regression method that is widely used to establish regression models [34,35]. The main principle of PLSR is to extract orthogonal factors of latent variables (LVs) and to establish regression relationships between the data set and the corresponding reference values. LVs are obtained by decomposing both independent and response variables. LS-SVM is a classical non-linear regression method for the computation of small sample data [36,37]. It uses a radial basis function kernel to map the input features into a high-dimensional feature space, thus converting a linear non-differentiable problem into a constrained quadratic programming problem. LS-SVM can search for potential patterns in the data and use the found patterns to predict the unknown data. On the other hand, for the establishment of classification models, the present study did not use the PLSR algorithm but the partial least squares discriminant analysis (PLS-DA) algorithm. The PLS-DA algorithm encodes the dependent variables of PLSR by means of dummy variables, thus enabling the description of different categories. The reference values of the dependent variables were set to -1 and 1 for intact and impact-damaged strawberries, respectively. For the LS-SVM algorithm, it also used −1 and 1 to represent intact and impact-damaged strawberries, respectively, and then built the classification models. The specific needs for building regression and classification models can be achieved by choosing different functions for the LS-SVM calculation.

For the accuracy of all regression models in this study, including the prediction of the extent of impact damage and the time of occurrence of the impact, they were evaluated mainly by the correlation coefficient of calibration (Rc), root-mean-square error of calibration (RMSEC), correlation coefficient of prediction (Rp), root-mean-square error of prediction (RMSEP), residual predictive deviation (RPD), and the absolute difference between RMSEC and RMSEP (AB_RMSE). Additionally, for the accuracy of the classification models, which was the identification of strawberries with impact damage, it was mainly evaluated by the correct rate, which was the ratio of the number of correctly identified samples to the total number of samples in the model. The best model and its corresponding best characteristic variable were determined based on the above indicators. All calculations for the multivariate data analysis were performed on MATLAB 2017b software (The MathWorks Inc., Natick, MA, USA).

### 2.5. Gas Chromatography-Mass Spectrophotometry (GC–MS) Analysis of VOCs

Strawberry samples (whole fruit) with different impact extents and storage times in three duplicates were analyzed by headspace solid-phase microextraction coupled to gas chromatography–mass spectrometry (HS–SPME/GC–MS). The GC–MS analysis was carried out using a 7890A gas chromatograph coupled to an Agilent 5975C mass spectrometer (Agilent Technologies, Santa Clara, CA, USA). Same as the e-nose measurement, before extracting VOCs, strawberry fruits were placed in a 100 mL glass beaker sealed by Parafilm PM996 for 30 min at 15 °C. Subsequently, the SPME fiber coated with 65 μm of polydimethylsiloxane and divinylbenzene (PDMS-DVB) (Supelco, Bellefonte, PA, USA) was inserted into the beaker to collect the VOCs. After 30 min of extraction, we inserted the VOC-adsorbed fiber into the injection pore, and it was desorbed at 240 °C for 5 min for the GC-MS measurement. Then, the VOCs were separated on a DB-WAX capillary column (30 m × 0.25 mm × 0.25 µm, J&W Scientific, Folsom, CA, USA). The main parameters were: high-purity helium as the carrier gas, with a flow rate of 1.0 mL/min; the initial column temperature was 40 °C, then increased to 100 °C at a rate of 3 °C/min, and then increased to 245 °C at a rate of 5 °C/min.

## 3. Results

### 3.1. Strawberries with Impact Damage

**Figure 1:**
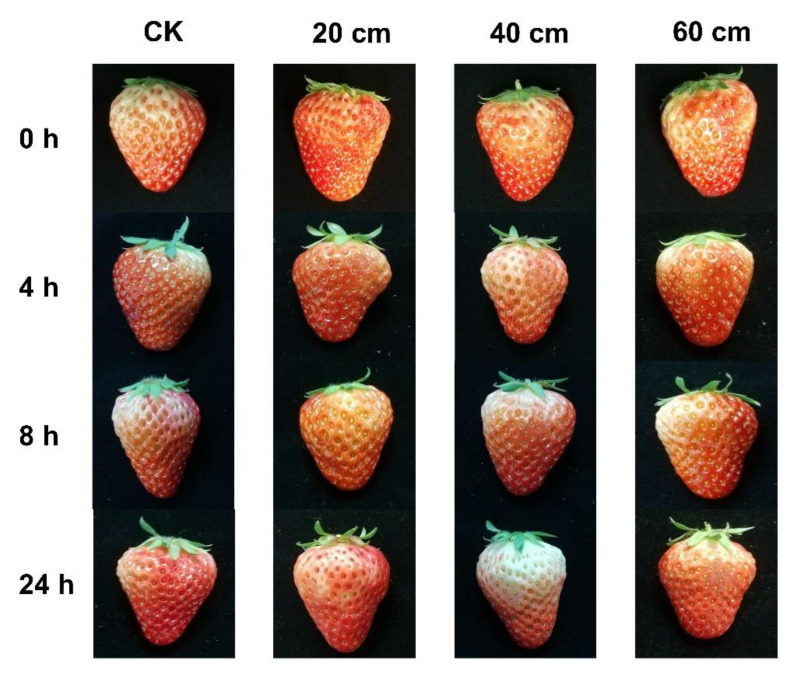
Strawberries with different damage extents and post-impact storage times.

Figure 1 shows the strawberries with different damage extents and post-impact storage times. As can be seen, it is difficult to tell from the pictures whether a strawberry has been mechanically damaged or not and to determine the extent of the damage. Even for strawberries with an impact extent of 60 cm, stored for 24 h after the impact, the damage was not easily detectable in appearance, except by very close observation or by pressing the wound by hand to feel the change in fruit firmness. Therefore, the next step was to investigate whether the VOCs of the strawberries had changed after the impact and whether they could be used for damage detection.

### 3.2. E-Nose Response of Strawberry

**Figure 2:**
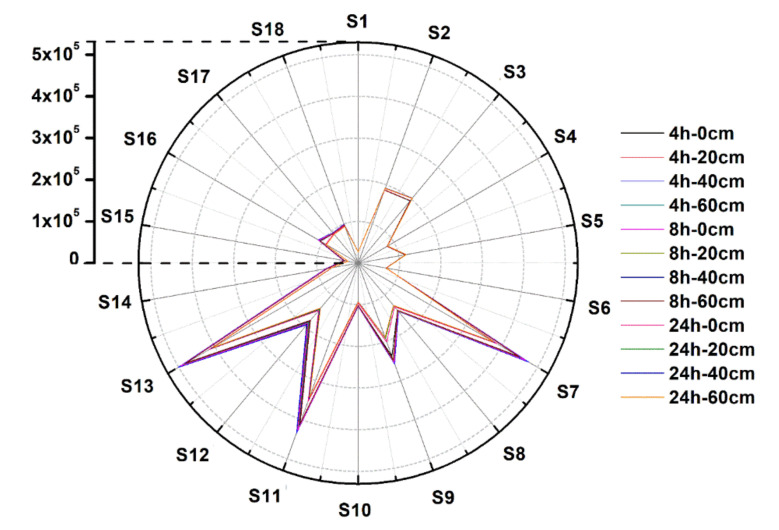
Polar plot of the average response data of the e-nose signal for strawberries that have suffered different extents of impact and have been stored for different times after the occurrence of the impact.

Figure 2 shows the average response data of the e-nose signal for strawberries that have suffered different extents of impact and have been stored for different times after the occurrence of the impact. In Figure 2, 18 e-nose sensors are represented by 18 angles from 0° to 340°, respectively. The spacing between each sensor is 20°. Specifically, the vectors with radii of angles 0° and 340° in Figure 2 represent Sensor 1 and Sensor 18, respectively. The upper left corner of the polar plot shows the scale of the sensor responses. Among them, Sensors 2, 3, 7, 9, 11, 12, and 13 had high response values. It can be seen that there are differences between the e-nose signals of the strawberries when they have been subjected to different extents of impact damage and stored for different periods of time, but these differences are difficult to distinguish by the naked eye in the figure. On the other hand, it should be noted that when more samples of e-nose data are displayed in the polar plot, there will be a serious overlap between the data, which makes it more difficult to perform direct visual analysis. Therefore, multivariate algorithms were used next to build models to achieve the detection of strawberry fruits that have suffered impact damage.

### 3.3. Prediction of the Extent of Impact Damage to Strawberries Falling from Different Heights

#### 3.3.1. Prediction of the Extent of Impact Damage in Strawberries at Different Times after Being Impacted

Predictive models for the extent of impact damage were developed based on EN_All_ and 11 characteristic variables of strawberries at different times (4, 8, 24 h) after suffering the impact, respectively. For the models established based on each time, there were a total of 96 samples: 72 samples for calibration and 24 samples for prediction. Since there are many models built based on different input variables, Table 1 only shows the best results obtained based on the two calibration algorithms. The RPD values for the PLSR models based on strawberry samples at 4 and 8 h after exposure to impact were 3.007 and 2.983, respectively. Nevertheless, the RPD value for the PLSR models was 1.377 for strawberry samples 24 h after impact, whose accuracy was worse than the models based on strawberry samples 4 and 8 h after impact.

The LS-SVM models obtained higher RPD values compared to the PLSR models. The average RPD value of all LS-SVM models was 3.642, which was 48.31% higher than that of the PLSR model (2.456). Particularly, the RPD value of the LS-SVM model, based on the samples 24 h after impact, was 2.6 times higher than that of the PLSR model. This illustrates that the LS-SVM algorithm was more suitable for the analysis of e-nose data in the present work, especially for the detection of strawberries that have been stored for a relatively long time after suffering an impact. The reason why it was more difficult to predict the extent of damage of the samples 24 h after the impact than the samples 4 and 8 h after the impact might be that strawberries with different extents of impact damage may change VOCs after storage for a period of time, and the difference in VOCs due to the impact becomes less obvious, so it will cause difficulty in predicting the extent of damage if the storage time after the impact is longer.

#### 3.3.2. Prediction of the Extent of Impact Damage in Strawberries at All Storage Times after Being Impacted

In practice, when e-nose technology is used to detect damaged strawberries, it is difficult to know how much time has passed since the damage occurred. Therefore, the models developed respectively based on strawberries at different storage times after the onset of impact can only be applied to some cases where it is known when the impact occurred. Therefore, other PLSR and LS-SVM models were established for the prediction of the extent of impact damage based on the strawberries at all storage times after being impacted, which means strawberries with different storage times after impact were all used for modeling. For the models established based all three times, there were a total of 288 samples: 216 samples for calibration and 72 samples for prediction. The results are also shown in Table 1.

When the PLSR algorithm was used to establish prediction models, the prediction results for both EN_All_ and the 11 characteristic variables were poor and could not be used for practical applications (data not shown). When the LS-SVM algorithm was used to establish prediction models, their prediction results were significantly better than the PLSR algorithm, except for the model based on the EN_Diff_ variable (data not shown). The best LS-SVM model achieved an RPD value of 2.730, which was 136.16% higher than the best PLSR model (1.156). Although the AB_RMSE values of the LS-SVM models were higher than those of the PLSR models, they did not exceed 0.3, except for the LS-SVM-EN_Diff_ model (data not shown), indicating that the robustness of the LS-SVM models was acceptable.

By comparing the model based on a certain storage time with the model based on all storage times, it can be seen that the models in the former had a significantly higher prediction accuracy. This suggests that models based on samples from different storage times obtained better prediction results than models based on strawberries from all storage times. The reason for this can be assumed to be that both the different extents of impact and the storage time after impact could cause changes in the VOCs of strawberries. For example, the change in VOCs of strawberries with a lighter impact extent after a long storage period may be similar to the change in VOCs of strawberries with a severe impact extent but a shorter storage period after the impact. Therefore, when strawberries with different storage times after impact were all placed in the same calibration sample set for calculation, it was more difficult than modeling strawberries based on different storage times after impact separately. The results of some previous studies also showed that it is more difficult to predict the extent of mechanical damage in fruits based on all storage times after suffering mechanical damage [38,39]. However, such difficulties could be compensated by using the LS-SVM algorithm to calibrate the prediction model. Especially considering that it is impossible to know the time of occurrence of the damage suffered by the fruit in the actual supply chain, the LS-SVM models developed in Table 1 are more suitable for practical applications.

### 3.4. Identification of Strawberries with Impact Damage

In practical applications, it is often not necessary to know the extent of mechanical damage suffered by the fruit, but only to determine whether the fruit has suffered mechanical damage during the supply chain. Therefore, a study was carried out to classify whether strawberries suffered impact damage based on their e-nose data. Discriminant models for mechanical damage detection were developed based on strawberries stored for 4, 8, and 24 h after impact and for all storage times, respectively. For the models established based on each time, there were a total of 96 samples: 72 samples for calibration and 24 samples for prediction. For the models established based all three times, there were a total of 288 samples: 216 samples for calibration and 72 samples for prediction. Models were built based on different input variables using PLS-DA and LS-SVM algorithms, respectively.

For strawberries at 4 h after impact, the correct rates of the discriminant models were basically above 90%, except for the LS-SVM-EN_Diff_ model. Among them, the LS-SVM-EN_All_, LS-SVM-EN_10_, and LS-SVM-EN_80_ models achieved 100% correct rates for both the calibration and prediction sets. For the strawberries at 8 h after impact, the results were similar to those of the models at 4 h after impact. The LS-SVM-EN_Max_, LS-SVM-EN_Sum_, LS-SVM-EN_60_, LS-SVM-EN_100_, and LS-SVM-EN_120_ models achieved 100% correct rates for both the calibration and prediction sets. For the strawberries at 24 h after impact, the accuracy of the discriminative models was lower than those of the models at 4 and 8 h after impact, when the PLS-DA algorithm was used for the calculation. However, when the LS-SVM algorithm was used for the calculation, the accuracy of the models was basically similar to those of the models for the first two time points. In addition, the LS-SVM-EN_Max_ and LS-SVM-EN_Sum_ models achieved 100% correct rates for both the calibration and prediction sets. When strawberries from all storage time points were used to establish the discriminant models, the average correct rate of the calibration and prediction sets for the PLS-DA models was 80.42%, while that of the LS-SVM models was 97.67% (excluding the LS-SVM-ENDiff model). In addition, the best models were LS-SVM-EN_Min_, LS-SVM-EN_Sum_, and LS-SVM-EN_10_, which had the same correct rates of 100% and 97.50% for calibration and prediction sets, respectively. Table 2 shows only the best results based on two calibration algorithms.

Analyzing the results of the models built based on different input variables, it can be found that for the models developed at the three time points, the numbers of models with 100% correct rates in both the calibration and prediction sets were 3, 6, and 2, respectively, which indicates that it is more favorable to discern whether the strawberry has suffered impact damage around 8 h after the impact, while it is more difficult to detect after 24 h. Nevertheless, when the LS-SVM algorithm was used to perform the calculation, the best models at all three time points achieved 100% correct rates (data not shown), indicating that if we only need to determine whether the strawberry is damaged by impact, then at least 4 h after the impact is sufficient for the detection.

When all three time points were used to build the discrimination models, no models reached 100% correct rates in the prediction set, which, again, indicates that detection would be more difficult if fruits with different storage times after suffering an impact are used for modeling. However, when the LS-SVM algorithm was used, the accuracy of the best model built based on all time points was already close to those of the models built based on individual time points. This shows a promising application of e-nose technology to detect whether strawberries have suffered mechanical damage during the supply chain.

### 3.5. Prediction of the Time of Occurrence of the Impact on the Fruit

Predicting when the fruit was impacted is important for both determining the cause of the impact and optimizing the supply chain process. Predictive models for the time of occurrence of the impact on the fruit were developed based on EN_All_ and 11 characteristic variables of strawberries at different impact extents (20, 40, and 60 cm), respectively. Both PLSR and LS-SVM algorithms were used to build models based on different input variables, respectively. For the models established based on each impact extent, there were a total of 96 samples: 72 samples for calibration and 24 samples for prediction. For the models established based on all impact extents, there were a total of 288 samples: 216 samples for calibration and 72 samples for prediction. Table 3 only shows the best results obtained based on two calibration algorithms.

The RMSEP values of the best PLSR models for strawberries subjected to impacts of 20 and 40 cm in height were around 3, which means that the model errors were, on average, 3 h; this is too large for practical applications. The RMSEP value of the best PLSR model for strawberries that suffered an impact of 60 cm in height was 1.135, which is an acceptable error for practical applications. Moreover, for the LS-SVM algorithm, the RMSEP of the best models based on different impact heights were all around 1, which is also acceptable. However, when all samples of all impact heights were used to build the PLSR and LS-SVM models, the RMSEP values increased substantially to 5.456 and 4.294, respectively, which means that the error of the models was, on average, 5 h. Considering the practical application is unable to know the specific extent of impact damage, the models based on all impact heights in Table 3 are more suitable for practical applications, and their results indicate that e-nose technology can only be used roughly to determine the time of impact occurrence.

### 3.6. GC-MS Analysis

GC-MS technology was used to analyze the changes of VOCs obtained in a non-destructive way after strawberries were damaged by impact. Analyzing the GC-MS data of nine groups of strawberry fruits with three extents of impact damage and three storage times after impact, 35 types of VOCs were identified, including 18 types of esters, 6 types of alkanes, 3 types of terpenes, 3 alcohols, and 5 other substances.

**Figure 3:**
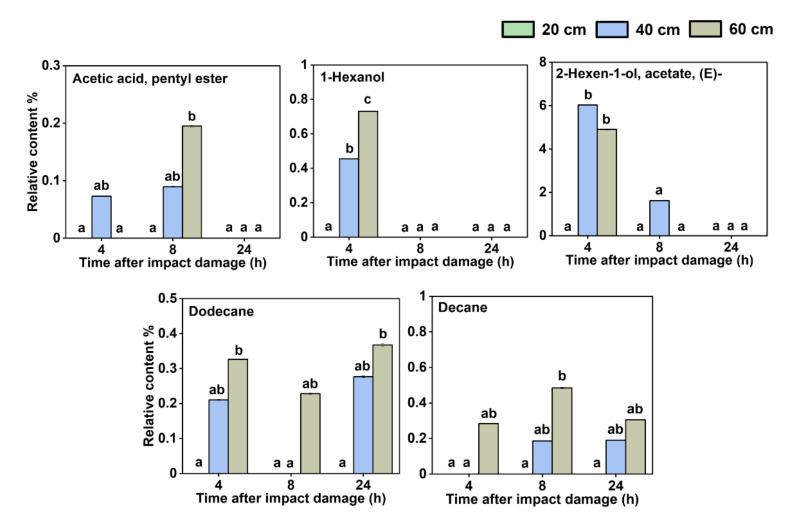
Five volatile organic compounds (VOCs) whose relative content was related to the extent of impact damage suffered by the fruit. Different letters (a, b, c, d) indicate significant differences (*p* < 0.05).

The relationship among the content of each VOC, the impact extent, and the storage time were further analyzed, and it was found that different impact damage extents and different storage times after impact caused some regular changes in the content of some VOCs. There were five VOCs whose relative content was related to the extent of impact damage suffered by the fruit (Figure 3), including acetic acid, hexyl ester, 1-hexanol, 2-hexen-1-ol, acetate, (E)-, dodecane, and decane. These VOCs were not detected when the impact height was 20 cm but were detected when the impact heights were 40 and 60 cm; the higher the impact height, the higher the substance content. However, some of them could be detected 4 and 8 h after impact but not detected at 24 h. This shows that these VOCs could be accumulated after the fruit is damaged by impact, but as the storage time increased after the impact, their content decreased, which may be related to the metabolic degradation of these VOCs in the fruit.

**Figure 4:**
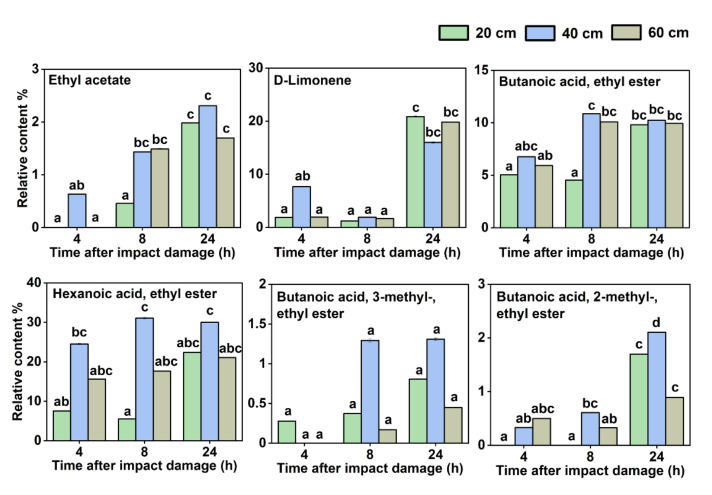
Six VOCs whose relative contents increased with the storage time after impact. Different letters (a, b, c, d) indicate significant differences (*p* < 0.05).

**Figure 5:**
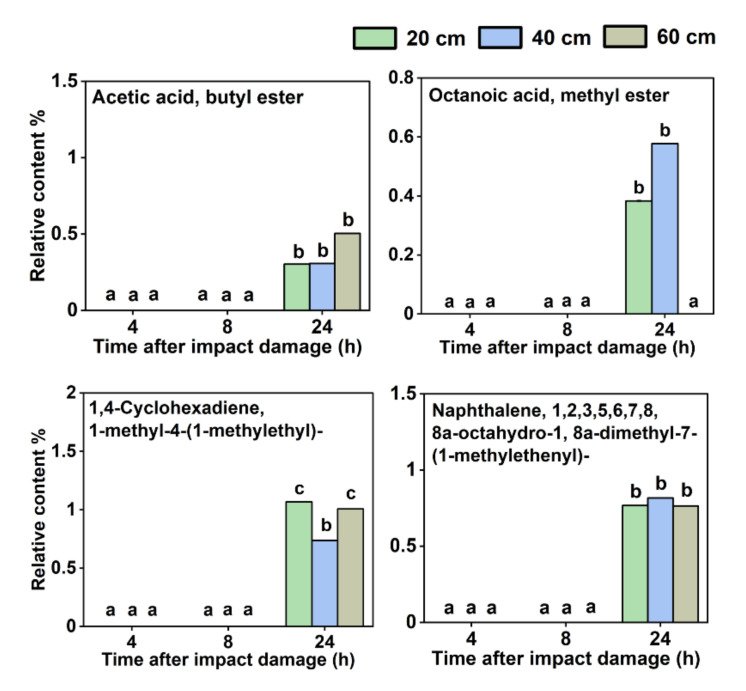
Four VOCs detected only in the later stage of storage after the fruit had been impacted. Different letters (a, b, c, d) indicate significant differences (*p* < 0.05).

Moreover, there were another six VOCs whose relative content increased with the storage time after impact (Figure 4), including ethyl acetate, D-limonene, butanoic acid, ethyl ester, hexanoic acid, ethyl ester, butanoic acid, 3-methyl-, ethyl ester, butanoic acid, 2-methyl-, and ethyl ester. Meanwhile, as shown in Figure 5, there were four VOCs, namely, acetic acid, butyl ester, octanoic acid, methyl ester, 1,4-cyclohexadiene, 1-methyl-4-(1-methylethyl)-, and naphthalene, 1,2,3,5,6,7,8,8a-octahydro-1,8a-dimethyl-7-(1-methylethenyl)-, which were not detected at 4 and 8 h after the impact but were detected as the storage time increased to 24 h.

**Figure 6:**
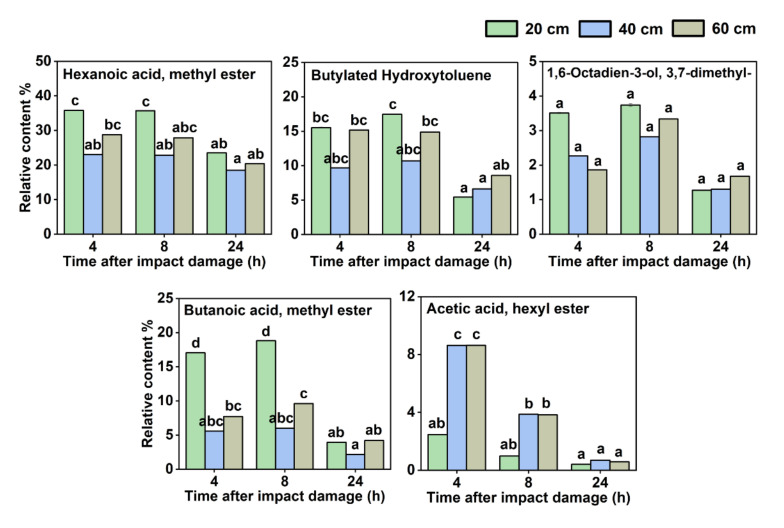
Five VOCs whose relative contents decreased with increasing storage time after impact onset. Different letters (a, b, c, d) indicate significant differences (*p* < 0.05).

In addition, Figure 6 illustrates five VOCs whose relative contents decreased with increasing storage time after impact onset, including hexanoic acid, methyl ester; butylated hydroxytoluene; 1,6-octadien-3-ol, 3,7-dimethyl-; butanoic acid, methyl ester; and acetic acid, hexyl ester. There were two other VOCs among them whose content changed in relation to the extent of impact damage the fruit suffered. Butanoic acid, methyl ester accumulated significantly more at the impact height of 20 cm than those at 40 and 60 cm, while acetic acid, hexyl ester accumulated significantly more at the impact heights of 40 and 60 cm than at 20 cm.

## 4. Discussion

Accurate detection of whether the fruit is damaged by mechanical forces or not could avoid the problem of reduced merchantability of the fruit product due to the inclusion of damaged fruit and also could prevent the damaged fruit from infecting other fruits around it. Therefore, the selection of strawberries that have suffered mechanical damage is important to guide distributors, retailers, and sellers to better decision-making on storage, transportation, and sales strategies. Particularly, since the packaging of fruits during the supply chain is commonly not completely sealed, a probe for detecting VOCs can be inserted into the packaging to collect VOCs from the fruits or detect VOCs at some openings of the packaging to determine whether the fruits have suffered mechanical damage based on VOCs.

Therefore, the detection of VOCs in the fruit is expected to enable the identification of those packaged strawberries that have suffered mechanical damage during the supply chain, resulting in good application prospects. Nevertheless, there are only a few studies using e-nose technology to detect mechanically damaged horticultural products. Demir et al. [40] used e-nose technology to detect blueberries with repeated impact; the correct classification rates ranged from 80% to 100%, and the cross-validation rates ranged from 75% to 100%. In another work, Ren et al. [41] classified the impact damage of apples using e-nose technology coupled with multivariate statistical analyses. The present work not only investigated whether e-nose technology could detect strawberries that have suffered impact damage but also further established and compared the results of models based on samples with different storage times after impact and samples with all storage times, thus making the developed models more realistic. However, the VOCs in fruits subjected to mechanical damage changed with longer storage times lead to greater difficulty in modeling based on samples with all storage times after impact. This is also well illustrated by the results in the present study. Meanwhile, in order to provide users with more data about the extent of damage to the fruit, the detection models for damage extent were also established through e-nose technology and obtained good prediction accuracy, which makes it possible to inform users of the specific extent of damage in addition to whether the fruit has suffered mechanical damage. Moreover, the present study evaluated whether e-nose technology could be used to predict when the impact damage had occurred. To our knowledge, relatively little work has been done for this purpose. The results will help us to know when the fruit suffers impact damage and determine why the impact occurred, which, in turn, will help to further optimize the fruit supply chain. In addition, it should be noted that the measurement process of e-nose signals of VOCs in the present study did not need us to destroy the strawberry fruits but was applied in a non-destructive way, so it is more suitable for application in the actual fruit supply chain process.

From the modeling results, it can be seen that when the impact occurred for 4 h, it was possible to determine the strawberry that has suffered impact damage and also to predict the extent of the damage. Of course, the detection was also possible when the impact occurred 8 and 24 h before. Further research is needed regarding the shortest detection time after the impact occurs. It should be noted, however, that if the time after the impact is too short to start the detection, the VOCs in the strawberry may not have changed enough to be detected.

In order to explain why the detection of VOCs by e-nose technology can be used to detect strawberries that have suffered mechanical damage from impact forces and to further investigate which VOCs were changed by the impact damage, this study further analyzed the VOCs of strawberries with impact damage using a non-destructive GC-MS technology. The results show that impact damage caused various changes in VOCs in strawberries. In particular, the relative contents of ethyl acetate and D-limonene were found to be increased with the increase of storage time after impact damage. Our previous research also found that the relative content of ethyl acetate in yellow peaches [39] and apples [42] was increased after being subjected to compression damage. It is proposed that ethyl acetate can be considered a potential volatile biomarker to detect damaged apples [42]. Regarding D-limonene, Chalupowicz et al. [43] indicated that limonene was a promising biomarker for the pathogen activity of citrus. Therefore, in the present study, ethyl acetate and D-limonene may be two important indicators for the detection of impact damage to strawberries. Studies on the VOCs changes in strawberries subjected to impact damage allow e-nose technology to detect damaged strawberries. Further research is needed to understand the biological mechanism underlying changes in certain VOCs in strawberries subjected to mechanical damage. In addition, to study the VOCs released by the whole fruit instead of the tissue homogenate, a non-destructive GC-MS method was applied to analyze the VOCs signals in the present study. The characteristic VOCs obtained by such non-destructive GC-MS analysis are more suitable for practical applications. It should be noted that the use of GC-MS technology was not considered in the present study to detect the damaged fruit in practical applications but to understand which VOCs of the fruit were changed as a result of the impact. Nevertheless, if the portability of the GC-MS detection instruments can be improved, then it may be possible to be applied in practice in the future as well.

There is still some work to be done if the fruit is to be judged by its VOCs in practical applications to determine whether it has suffered mechanical damage. The first is that the fruit will suffer from the synergistic effects of multiple mechanical damages during the supply chain, such as vibration, extrusion, impact, and friction. These mechanical damages lead to possible differences in the characteristic VOCs. Additionally, it is unknown whether there is a synergistic effect between multiple mechanical forces on the change of VOCs. Therefore, it is necessary to further investigate the characteristic VOCs of other mechanical forces and determine the best characteristic VOCs for the detection. The second is that there are many factors that affect VOCs in fruits, such as variety, cultivation environment and method, harvest year, and storage conditions. Therefore, it is necessary to collect more comprehensive sample information to build into the detection model. The third is the need to research and screen some characteristic VOCs of mechanical damage and develop their sensors so as to find the damaged fruit more accurately. The fourth is that the detection equipment must be miniaturized. Large-scale e-nose instruments used in laboratories are not suitable for practical industrial applications. Some portable or small e-nose instruments have been developed [44,45]. They are expected to be applied to the detection of VOCs in fruits in practice in the future. The fifth is that the cold chain is a common method in the fruit supply chains. However, low temperatures will slow down the release of VOCs from the fruit. Therefore, in the cold chain environment, more powerful VOC analysis technology is needed to detect damaged fruits.

## 5. Conclusions

The results of the present study show that the use of e-nose technology to measure the VOCs in strawberries can be used to detect the fruits damaged by impact force. For example, it is possible to directly detect impact-damaged strawberry fruits. Even when the models were calibrated based on all strawberries at 4, 8, and 24 h after the impact, the correct rate of the prediction set could still be up to 97.5%, while 100% correct rates were achieved when the modeling was based on strawberries at different times after the impact. Additionally, e-nose technology can further predict the extent of impact damage. The highest RPD values of 3.548, 3.764, and 3.614 were achieved for samples stored for 4, 8, and 24 h after impact, respectively, while the highest RPD of 2.730 was obtained for all samples stored after the impact, thus demonstrating the powerful ability of e-nose technology to detect mechanical damage to strawberries. In addition, the present study evaluated whether the e-nose technology could be used to predict the time of impact on strawberries. The results show that if strawberries with different impact extents were considered separately, then the models could predict the time after impact. However, if strawberries with different impact extents were used together in the modeling, then the time prediction was difficult. Moreover, the results show that the LS-SVM algorithm was more suitable for modeling than the PLSR algorithm in detecting strawberries that have suffered impact damage. In addition to e-nose technology, this study further analyzed the main characteristic VOCs that have changed due to impact force through GC-MS technology, which is the basis for the detection of damaged fruits by e-nose technology. This study provides a new method for the rapid detection of mechanical damage to fruits during the supply chain through the use of technologies that can non-destructively detect VOCs in fruits, such as e-noses.

## Figures and Tables

**Table 1:** Best models for the prediction of the extent of impact damage in strawberries at different storage times (4, 8, and 24 h) after being impacted and all kinds of storage times. Correlation coefficient of calibration (Rc), root-mean-square error of calibration (RMSEC), correlation coefficient of prediction (Rp), root-mean-square error of prediction (RMSEP), residual predictive deviation (RPD), and the absolute difference between RMSEC and RMSEP (AB_RMSE).

Time	Feature Variables	Calibration Method	Calibration			Prediction				AB_RMSE
			R_c_	Rc^2^	RMSEC	R_p_	Rp^2^	RMSEP	RPD	
4 h	EN_Sum_	PLSR	0.944	0.892	0.372	0.944	0.858	0.426	3.007	0.053
4 h	EN_60_	LS-SVM	0.995	0.989	0.117	0.960	0.918	0.323	3.548	0.206
8 h	EN_All_	PLSR	0.936	0.876	0.388	0.945	0.873	0.407	2.983	0.019
8 h	EN_All_	LS-SVM	0.986	0.972	0.185	0.964	0.929	0.304	3.764	0.119
24 h	EN_100_	PLSR	0.837	0.701	0.634	0.688	0.434	0.821	1.377	0.187
24 h	EN_Sum_	LS-SVM	0.990	0.980	0.165	0.962	0.920	0.309	3.614	0.144
All	EN_All_	PLSR	0.648	0.420	0.858	0.510	0.250	0.982	1.156	0.124
All	EN_All_	LS-SVM	0.993	0.984	0.143	0.931	0.858	0.428	2.730	0.285

**Table 2:** Best models for identification of strawberries with impact damage.

Feature Variables	Calibration Method	4 h		8 h		24 h		all	
		Calibration	Prediction	Calibration	Prediction	Calibration	Prediction	Calibration	Prediction
EN_All_	PLS-DA	98.3%	100.0%	95.0%	94.1%	95.0%	85.3%	96.1%	92.1%
EN_Sum_	LS-SVM	100.0%	97.0%	100.0%	100.0%	100.0%	100.0%	100.0%	97.5%

**Table 3:** Best models for the prediction of the time of occurrence of the impact on the fruit.

High	Feature Variables	Calibration	Calibration			Prediction				AB_RMSE
			R_c_	Rc^2^	RMSEC	R_p_	Rp^2^	RMSEP	RPD	
20 cm	EN_100_	PLSR	0.967	0.935	2.183	0.945	0.892	2.903	3.056	0.720
20 cm	EN_Max_	LS-SVM	1.000	1.000	0.000	0.996	0.991	0.823	10.743	0.823
40 cm	EN_Min_	PLSR	0.883	0.779	4.069	0.925	0.792	3.667	2.508	0.402
40 cm	EN_All_	LS-SVM	1.000	1.000	0.101	0.995	0.983	1.056	8.704	0.955
60 cm	EN_All_	PLSR	0.993	0.986	1.035	0.991	0.982	1.135	7.487	0.100
60 cm	EN_Sum_	LS-SVM	1.000	1.000	0.000	0.998	0.995	0.609	13.944	0.609
all	EN_sum_	PLSR	0.799	0.638	4.692	0.755	0.568	5.456	1.523	0.764
all	EN_All_	LS-SVM	0.932	0.856	2.957	0.876	0.733	4.294	1.969	1.337

## Data Availability

Not applicable.
